# Predictors of antipsychotics switching among ambulatory patients with schizophrenia in Ethiopia: a multicenter hospital-based cross-sectional study

**DOI:** 10.1186/s12991-023-00472-z

**Published:** 2024-01-03

**Authors:** Mekdes Kiflu, Telake Azale, Kale Gubae, Samuel Agegnew Wondm, Ephrem Mebratu, Asrat EliasErgena, Ousman Abubeker, Gizework Alemnew Mekonnen

**Affiliations:** 1 Unit of Clinical Pharmacy, Department of Pharmacy, College of Health Science, Debre Markos University, Debre Markos, Ethiopia; 2 Department of Health Education and Behavioral Science, Institute of Public Health, College of Medicine and Health Science, University of Gondar, Gondar, Ethiopia; 3 Unit of Pharmacology, Department of Pharmacy, College of Health Science, Debre Markos University, Debre Markos, Ethiopia; 4 Department of Pharmaceutical Chemistry, College of Medicine and Health Sciences, University of Gondar, Gondar, Ethiopia; 5 Department of Clinical Pharmacy, School of Pharmacy, College of Medicine and Health Science, University of Gondar, Gondar, Ethiopia

**Keywords:** Schizophrenia, Determinants, Antipsychotics switch, Switching strategies

## Abstract

**Introduction:**

A change of therapy from one to another antipsychotic medication is currently the main challenge of therapy. This study aimed to assess the prevalence of antipsychotic medication switches and determinants among patients with schizophrenia in Northwest Ethiopia.

**Methods:**

Multi-center hospital-based cross-sectional study was conducted at five Comprehensive Specialized Hospitals found in Northwest Ethiopia from April 30, 2021, to August 30, 2021. Data were extracted from both patients’ medical charts and interviews. Data were entered into Epi-data software version 3.5.1 and exported to SPSS version 25.0 for analysis. A multivariable logistic regression model was fitted to identify factors associated with medication regimen switch. The level of significance of the study was kept at a *p*-value of 0.05 with a 95% confidence interval.

**Result:**

A total of 414 patients are involved in the study, and 188 (45.5%) of patients switched antipsychotics within one year. The unavailability of the medication is the commonest reason for switching. Being male [AOR = 2.581, 95% CI (1.463, 4.552)], having relapse [AOR = 2.341,95% CI (1.169,4.687)], history of hospitalization in the past year [AOR = 3.00,95% CI (1.478,5.715)] and taking typical antipsychotics [AOR = 3.340, CI (1.76, 6.00)] had a significant association with antipsychotics switching.

**Conclusions and recommendations:**

There is a high prevalence of antipsychotic switches among schizophrenia patients. Prescribers need to be careful while dosing, selecting, and switching antipsychotics, hence may help reduce discontinuation and unnecessary switch and thus achieve optimal clinical management.

## Background

Schizophrenia is a chronic, disabling heterogeneous group of brain disorders with yet an unknown etiology and a poorly understood pathophysiology [1]. It is a severe form of mental illness affecting 20 million people [2] and it is the 12th most disabling ailment worldwide [3]. Antipsychotics are the first-line agents for managing the disease condition. As with other chronic illnesses, clinicians must decide which medication to take and whether to switch a patient’s current medication to improve treatment response, reduce intolerable side effects and improve quality of life and functioning [4].

Antipsychotic switching (AS) is changing one antipsychotic to another and it is a common clinical practice. It is widely believed that patients who do not respond to one member of a psychotropic drug class or who experience troublesome side effects may have a better response to another agent in the same or similar class [5]. Because of presumed variability in individual responsiveness, this has been thought to be true, even when the new drug is not superior to the old drug for that outcome or side effect in head-to-head comparisons [6]. But, in any of the cases, the practice should be carried out cautiously and under close observation of the clinicians [7].

Studies showed that there is an increased risk of destabilization and clinical deterioration among patients who switched antipsychotics when they are compared to those who stayed on their former regimen [8]. Furthermore, the patients who had to switch medications were more likely to discontinue their medication than those who were assigned to stay on the medication they entered with [9]. Discontinuation syndromes, changes in psychopathology, pharmacodynamics interactions, pharmacokinetic interactions, and neuroleptic malignant syndrome are some other reported problems associated with AS [10].

Antipsychotic medication switching is quite limited in its success and it does not produce the expected results, with considerably poorer clinical and economic outcomes and higher incidence of treatment interruption accrued an additional 25% increase in annual total health care costs per patient related to acute care expenditures [11, 12]. With all these drawbacks and uncertainties, an antipsychotic switch is being increased over time [13–17]. Reports showed that the patients’ AP medications were switched seven times over one year period, with the mean number of antipsychotic switches being 2.1 [18].

Understanding the actual burden of antipsychotic switching has not been easy. Empirical data on the prevalence, patterns, predictors, and strategies of antipsychotic medication switches in schizophrenic patients in low and middle-income countries such as Ethiopia with poor mental health services were lacking [19]. Knowing the burden and determinants of antipsychotic switching will be crucial to understanding and monitoring the care given to these vulnerable populations, guiding important clinical and policy decision-making, and identifying patients at higher risk of the antipsychotic switch, hence decreasing morbidity, mortality, and clinical costs. Although there is a considerable number of schizophrenia patients on APs treatment at five comprehensive Specialized Hospitals, in Northwest Ethiopia to date there have been no published studies determining the burden and predictors for regimen switches among schizophrenic patients. This study aimed to determine the prevalence of antipsychotic switches and factors associated with antipsychotic switches among schizophrenic patients.

## Methods

### Study design, setting, and period

A hospital-based cross-sectional study was conducted at psychiatry ambulatory care of five Comprehensive specialized hospitals in Northwest Ethiopia, namely Felege Hiwot Comprehensive Specialized Hospital (FHCSH), Tibebe Ghion Comprehensive Specialized Hospital (TGCSH), Debre Markos Comprehensive Specialized Hospital (DMCSH), University of Gondar Comprehensive Specialized Hospital (UoGCSH), and Debre Tabor Comprehensive Specialized Hospital (DTCSH) from April 30, 2021, to July 30, 2021. All the hospitals included in the study provide inpatient psychiatry services for those who are admitted psychiatric patients and chronic follow-up psychiatric clinics for ambulatory patients.

### Study population

Schizophrenia patients who came to the chronic follow-up clinic during the study period.

### Inclusion and exclusion criteria

Adult (age greater than 18 years) schizophrenic patients who had received therapy at one of the five comprehensive specialized hospitals for at least a year and who have the insight to respond to oral questions (using insight assessment tool [20]) were included in the study, while patients who had an incomplete follow-up data on most pertinent variables on charts were excluded.

### Sample size determination and sampling techniques

The sample size was determined using a single population proportion formula. Taking into account the following assumption:

*p* = the prevalence of antipsychotic switches, 42.8% in a previous study done in Addis Ababa, Ethiopia [21]. The margin of error (*α*) was 0.05; the level of confidence (95%), 1.96 Z (standard normal distribution), and 10% non-response rate, the final sample size became 414.

A total of 3709 patients with a clinical diagnosis of schizophrenia were on follow-up in five of the study sites. These schizophrenic patients came into the hospital at a maximum of every three months to refill their antipsychotic medications. Proportional allocation of the participants was done at each hospital and with a total sample size of 414. A systematic random sampling technique was used to select study participants with a sampling fraction k of 9. The starting point was selected by the lottery method from numbers 1 to 9. Then, every 9th patient was interviewed and their medical records were reviewed. About 77 participants were selected from DMCSH (*N* = 686), 126 from FHCSH (*N* = 1133), 43 from TGCSH (*N* = 380), 46 from DTCSH (*N* = 410), and 122 from UoGCSH (*N* = 1100).

### Data collection and quality control technique

The data collection tool was developed after reviewing published literature. The pretest was done in 5% (21 patients) of the sample size before conducting the study and the findings of the pretest were not included in the final analysis. The necessary amendment was done to the final version of the questionnaire during the process of the pretest. The questionnaire for the interview contained socio-demographic characteristics, MARS (Medication Adherence Rating Scale), OSSS (Oslo Social Support Scale), and medication-related questions. The document review was done using a data extraction format to collect the data related to clinical factors; medication-related factors and reasons for medication switch. The data were collected by five trained psychiatry nurses under daily supervision.

To ensure the quality of the data training was given to data collectors and an English version of the data collection questionnaire was translated to Amharic and back translated to English, and the data gathering tool was sent to a senior physician with psychiatry specialty face validity and approval.

### Data processing and analysis

Data were coded and cleaned using EpiData version 3.5.1 and exported to SPSS version 25 for analysis. Descriptive statistics were used to present the socio-demographic and behavioral factors of the participants. We used percentages, mean, standard deviation, frequencies, and cross tabulation to describe patient characteristics. A chi-square test was done on categorical variables and bivariable logistic regression was done and the predictor variable which had a significant association with the predicted variable at *p*-value < 0.25 in the bivariable logistic regression model was selected. A multivariable logistic regression model was fitted to identify factors associated with antipsychotic medicine switch. In the multivariable logistic regression analysis, variables with *p* ≤ 0.05 at 95% CI were considered statistically significant.

### Ethical consideration

The study was conducted by following declaration of Helsinki. Ethical clearance was obtained from the School of Pharmacy, Department of Clinical Pharmacy Ethical Review Committee, University of Gondar, Gondar with a reference number SOP/483/2021. Informed verbal consent, which is applicable in the study setting was obtained from participants, after the data collectors presented the purpose of the study, why and how they are selected to be involved in the study, what is expected from them, and that they can withdraw from the study at any time. The name and addresses of the patients were not recorded in the data abstraction formats, and data were collected unanimously to ensure confidentiality.

### Operational definitions

#### Relapse

Re-emergence or aggravation of psychotic symptoms [22].

#### Substance use history

Indicates using Khat, cigarettes, and tobacco within 3 months.

#### Switching

Any change of therapy from one oral antipsychotic to another antipsychotic.

#### Abrupt switching

Abrupt cessation of the current drug, with the abrupt introduction of the new one at the expected therapeutic dosage.

#### Cross-tapering switching

Slow downward adjustment of the dosage of the current medication, with slow upward adjustment of the dosage of the new drug.

#### Adherence

The degree to which the person's medication-taking behavior specifically refers to the extent to which a patient follows the mutually agreed treatment plan [23, 24].

#### Adherent

Medication adherence rating scale score of six and above.

#### Non-Adherent

Medication adherence rating scale score of less than six.

#### The chlorpromazine dose equivalencies (CPZeq)

A measure of antipsychotics' respective potencies.

#### Sub-therapeutic dose, optimal dose, high dose, and very high dose

A CPZeq maintenance dose of less than 300 mg, a dose between 300 and 600 mg, a dose between 600 and1000 mg, and a dose beyond 1000 mg, respectively.

#### Disability

A restriction or inability to perform an activity in the manner or within the range considered normal for a human being, mostly resulting from the impairment [25].

#### No disability, mild disability, moderate disability, severe disability, and extreme disability

WHODAS score between 0 and 4%, 5 and 24%, 2 and 49%, 50 and 95%, and 96 and 100%, respectively [26].

#### Strong social support, moderate social support, and poor social support

OSSS-3 sum score between 12and 14, between 9 and 11, and between 3 and 8, respectively [27].

#### No insight, partial insight, and full insight

Tool a total score of 0, 1–2, and 3, respectively, using insight assessment score [20].

## Results

### Socio-demographic and behavioral characteristics of the respondents

A total of 414 schizophrenic patients were enrolled in this study. The results showed that 220 (53%) were males and around one-third of the patients age lie between 25 and 34 years. A large proportion of the participants, 169 (40.8%) had no formal education, and more than half 244 (58.9%) lived in rural areas. Most of the participants (366 (58.9%)) were Orthodox religious followers. Additionally, a large proportion of the participants were single 174 (42%) (Table 1).

**Table 1:** Sociodemographic and behavioral characteristics of ambulatory patients with schizophrenia in five comprehensive specialized hospitals, Northwest Ethiopia, 2021 (*n* = 414)

Baseline characteristics	Category	Frequency (%)
Sex	Male	220 (53.1)
	Female	194 (46.9)
Age	≤ 24	96 (23.2)
	25–34	129 (31.2)
	35–44	124 (30.0)
	45–54	43 (10.4)
	≥ 55	22 (5.3)
Educational Status	No formal education	169 (40.8)
	Primary education	90 (21.7)
	Secondary education	87 (21.0)
	College & above	68 (16.4)
Religion	Orthodox	366 (88.4)
	Muslim	43 (10.4)
	Protestant	5 (1.2)
Occupation	Farmer	127 (30.7)
	Housewife	61 (14.7)
	Student	58 (14.0)
	Unemployed	57 (13.8)
	Gov’t employer	56 (13.5)
	Private business	44 (10.6)
	Daily laborer	11 (2.7)
Marital status	Single	174 (42.0)
	Married	166 (40.1)
	Divorced	69 (16.7)
	Widowed	5 (1.2)
Living arrangements	With relatives/families	353 (85.3)
	Alone	51 (12.3)
	In charitable organization	10 (2.4)
Residence	Rural	244 (58.9)
	Urban	170 (41.1)
Cigarette smoking	No	377 (91.1)
	Yes	37 (8.9)
Alcohol use	No	350 (84.5)
	Yes	64 (15.5)
Cannabis use	No	411 (99.3)
	Yes	3 (0.7)
Khat use	No	398 (96.1)
	Yes	16 (3.9)

### Clinical characteristics of the respondents

The present study revealed that two-thirds of the study participants, 276 (66.66) had been ill for more than 10 years. The frequency of their follow-up was every month for 175 (42.3%) of the patients followed by every two months for 128 (30.90%) of the patients. The majority of the participants, (93.2%), had no comorbidity. About 241 (58.2%) of patients had a history of hospital admission and 185 (28%) of the patients experienced a relapse in the past year (Table 2).

**Table 2:** Clinical characteristics of ambulatory schizophrenia patients in five comprehensive specialized hospitals, Northwest Ethiopia, 2021 (*n* = 414)

Clinical characteristics	Category	Frequency (%)
Duration of illness (years)	1–10 years	138 (33.33)
	11–20 years	205 (49.51)
	21–30 years	71 (17.15)
Follow-up	Monthly	175 (42.3)
	Every 2 month	128 (30.9)
	Every 3 month	100 (24.15)
	Every 4 months or Above	11 (2.7)
Comorbid Mental illness	No	386 (93.2)
	Yes	28 (6.8)
Type of comorbid mental illness (*n* = 28)	Substance Use Disorder	13 (46.4)
	Anxiety Disorder	9 (32.2)
	Major Depressive Disorder	6 (21.4)
Comorbid medical illness	No	400 (96.6)
	Yes	14 (3.4)
Type of Medical illness (*n* = 14)^c^	Diabetic Mellitus	4 (28.6)
	Hypertension	3 (21.4)
	Others^a^	7 (50.0)
Medication used for comorbid medical illness (*n* = 14)^c^	Enalapril	3 (21.43)
	Metformin	2 (14.29)
	TDF + 3TC + DTG	2 (14.29)
	Insulin	2 (14.29)
	Lasix	2 (14.29)
	Others^b^	4 (28.56)
Medication used for other comorbid mental illnesses (*n* = 28)	Amitriptyline	13 (46.43)
	Fluoxetine	13 (46.43)
	Imipramine	1 (3.57)
	Sertraline	1 (3.57)
Relapse	No	229 (55.3)
	Yes	185 (44.7)
Number of relapses (*n* = 185)	Once	150 (81.1)
	Twice	33 (17.8)
	Three times	2 (1.1)
Admission	Yes	241 (58.2)
	No	173 (41.8)
Number of admissions	Once	193 (80.3)
	Twice	47 (19.1)
	Three times	1 (0.6)

^a^Epilepsy, HIV/AIDS, Heart failure

^b^Nifidipine, phenytoin, phenobarbital, Amlodipine

^c^More the one answer

### Medication-related characteristics and level of social support of the participants

Using the MARS tool we confirmed that the majority 330 (80%) of the participants are adherent to their medication. More than half, 252 (60.9%) of the study participants had community-based health insurance and received their medication through this system, and most, 384 (92.8%) participants didn’t use any adjuvant medications. Troublesome side effects while taking antipsychotics were not common in the majority, 364 (88%) of the participants. Furthermore around half 210 (50.7%) of the participants had moderate social support (Table 3).

**Table 3:** Medication-related characteristics and level of social support of ambulatory schizophrenia patients in five comprehensive and specialized hospitals, Northwest Ethiopia, 2021 (*n* = 414)

Variable	Category	Frequency (%)
Adherence	Adherent	330 (80)
	Non-adherent	84 (20)
Source of medication fee	Community-based health insurance	252 (60.9)
	Out of a pocket purchase	162 (39.1)
Combined medication use	None	384 (92.8)
	Benzhexol	20 (4.8)
	Diazepam	8 (1.9)
	Clonazepam	2 (0.5)
Side effects	Yes	50 (12)
	No	364 (88)
Level of social support	Poor	133 (32.1)
	Moderate	210 (50)
	High	71 (17)

### Prevalence and frequency of antipsychotic switching

The present study showed that around 45.4% [95% CI (40.8—49.8)] of the participants had experienced an antipsychotic medicine switch in the past 12 months, and among them, 171 (91.0) of the patients had switched once in this specified period. Most of the APs switch was done before the drug was administrated at the optimal dose, 386 (93.2%) of the participants had taken sub-therapeutic doses of antipsychotics before switching to another antipsychotic. The study participants from DTCSH and UoGCSH were with a higher frequency of antipsychotic switching, 52.1% and 51.6%, respectively (Table 4).

**Table 4:** Practice and frequency of antipsychotic switching in patients with schizophrenia patients at the ambulatory ward of five comprehensive specialized hospitals, Northwest Ethiopia, 2021 (*n* = 414)

Variables	Category	Frequency (%)
Antipsychotic switch	Yes	188 (45.4)
	No	226 (54.6)
Frequency of switching	Once	171 (91)
	Twice	17 (9)
Maintenance dose before switching	Sub-therapeutic	386 (93.2)
	Optimum	27 (6.5)
	High	1 (0.2)
Antipsychotic switch with the study sites	UoGCSH	
	Had switching	63 (51.6)
	No switching	59 (48.4)
	DMCSH	
	Had switching	27 (35)
	No switching	50 (75)
	FHCSH	
	Had switching	57 (45)
	No switching	70 (35)
	DTCSH	
	Had switching	25 (52.1)
	No switching	23 (47.9)
	TGCSH	
	Had switching	16 (40)
	No switching	24 (60)

UoGCSH: University of Gondar Comprehensive Specialized Hospital; DMCSH: Debre Markos Comprehensive Specialized Hospital; FHCSH: Felege Hiwot Comprehensive Specialized Hospital; DTCSH: Debre Tabor Comprehensive Specialized Hospital; TGCSH: Tibebe Ghion Comprehensive Specialized Hospital

### Reason and strategies used for switching

From a total of 188 patients who had antipsychotics switch, unavailability of the medication was the most commonly (51.7%) reported reason for APs switch followed by an inadequate or partial response to the existing medication (14.1%). Furthermore, our study revealed that APs switch was done abruptly among 98.40% of the respondents who had switched to another APs for the first time within one year but all switches were done abruptly without titration for those who had APs switched for the second time (Table 5).

**Table 5:** List of reasons for switching in ambulatory schizophrenia patients at five comprehensive specialized hospitals, Northwest Ethiopia, 2022; (*n* = 188)

Variable	Category	Frequency (%)
Reasons for switching	Unavailability of medicines	106 (51.7)
	Inadequate/partial response	29 (14.1)
	Presence of side effects	27 (13.2)
	Unrecorded reason	18 (8.8)
	Non-adherence	14 (6.9)
	Others^a^	11 (5.3)
Primary switching	Abrupt switching	185 (98.4)
	Cross-titration switching	3 (1.6)
Secondary switching	Abrupt switching	17 (100)

^a^Frequent relapse, Cost of the medication, Resistance to the existing medication

### Factors associated with antipsychotic medication switching

The multivariable logistic regression analysis was fitted to identify factors associated with the antipsychotic medication switch. Being male sex, history of relapse, previous history of admission, and taking typical antipsychotic medicines showed statistically significant association with antipsychotic medicine switch (Table 6). The odds of APs switch in males had 2.58 times higher than the antipsychotic switch in females [AOR = 2.58, 95% CI (1.46, 4.55)]. The odds of antipsychotic switch in patients who had a relapse had 2.34 times higher than patients without relapse [AOR = 2.34, 95% CI (1.17, 4.69)], and patients who had a hospital admission in the past year had 3 times higher odds of switching antipsychotics than patients who didn't have admission history [AOR = 3.00, 95% CI (1.48, 5.72)]. The odds of antipsychotic switch in patients who were on typical antipsychotics was 3.34 times higher than in patients on atypical antipsychotics [AOR = 3.34, CI (1.77, 6.00)].

**Table 6:** Predictors of antipsychotic medicine switch in ambulatory patients with schizophrenia at five comprehensive specialized hospitals, Northwest Ethiopia, 2021 (*n* = 414)

Variable	Category	Antipsychotic switch		COR (95% CI)	AOR (95% CI)	*p*-value
		Yes (%)	No (%)			
Gender	Male	116 (26)	104 (46)	1.89 (1.27–2.80)	2.58 (1.46–4.55)	0.001**
	Female	72 (38.3)	122 (54)	1	1	
History of admission in the past 12 months	Yes	139 (74)	102 (45.1)	3.44 (2.27–5.23)	3.00 (1.47–5.71)	0.002**
	No	49 (26)	124 (54.9)	1	1	
Groups of medication in the past 12 months	FGAs	140 (74.5)	86 (38)	4.74 (3.22–7.44)	3.34 (1.76–6)	0.000***
	SGAs	48 (25.5)	140 (62)	1	1	
History of relapse in the past 12 months	Yes	76 (40)	109 (48.2)	0.73 (0.32–0.75)	2.34 (1.16–4.68)	0.016*
	No	112 (60)	117 (51.8)	1	1	

## Discussion

### Prevalence of antipsychotic switching

The present study showed that the prevalence of antipsychotics switch was high. Being male, having a history of relapse, previous history of admission, and use of conventional (first generation) antipsychotics showed statistically significant association with antipsychotic switch. Most switches were done abruptly and unavailability of the medication was the most commonly reported reason for APs switch.

In the present study, the prevalence of antipsychotic drug switches was 45.4%. This finding is in line with a report from Amanuel Mental Specialized Hospital (42.8%) [21] and the Netherlands (48.5%) [28]. On the other hand, the current report was higher as compared to studies from the USA 14% to 33% [13, 29, 30], Canada (11%) [31], Japan (27.4%) [32], and South Africa (34%) [33]. A possible explanation for these discrepancies could be due to the smaller sample size used by the later studies and most of the studies focused on specific atypical antipsychotics which had a lower rate of switching. Furthermore, the unavailability of most of the effective drugs used to treat schizophrenia in our resource-limited settings could increase the switching.

The present study revealed that the most common reason for APs switch was the unavailability of the medicines. Unavailability and interrupted supply of medication is the most common reason for discontinuation as well as switching of medication in Ethiopia. This problem affected the mental health service in the country [21, 34]. The concern was also increased due to the COVID-19 outbreak which made the international pharmaceutical supply chain to disrupted and caused serious ramifications for global medicine access, particularly in low- and middle-income countries (LMICs) [35, 36]. In contrast, a prospective observational study done in Slovenia showed that the most common causes for switching antipsychotics were adverse reactions and inefficacy or lack of efficacy [37].

Inadequate or partial response was the other reason for antipsychotic medicine switching in the present study. The same reason for antipsychotic medicine switching has been reported by different researchers in different setups [5, 38–44]. So proper use of antipsychotic medicine by clinicians and patients according to national and international guidelines is recommended to maintain the effective use of the medications.

Being male had a 2.58 times higher risk of APs medicine switch than females. This finding is inconsistent with studies done in the USA [12, 17]. The reason why APs switch was more frequent in males might be concomitant substance use disorder is more common in males than females at which patients with drug use disorders were more likely to switch antipsychotic medications [45] furthermore, males had a lower response to antipsychotic medicines than females due to the lipophilic properties of neuroleptics, females are exposed for lower doses of antipsychotics for treatment than males. As a result, male patients are exposed to higher antipsychotic doses, resulting in unwanted side effects and medication non-adherence. Male schizophrenic patients had a higher rate of non-remission than female schizophrenic patients, and men were hospitalized more frequently and for longer periods than female schizophrenic patients, making males particularly vulnerable to switching [5, 46].

The odds of an antipsychotic medication switch had 2.34 times more frequent in those patients who had a relapse in the previous year than in those who had no relapses. This finding is consistent with another study done in Ethiopia [21]. The reason might be that in patients with relapse, there is a worsening of positive and negative symptoms which leads to a change in antipsychotic medication related to the worsening of the illness [47–51]. In addition to the aforementioned reason, patients with schizophrenia experienced progressive brain tissue loss after onset implying that relapse may aggravate tissue loss in several brain regions resulting in medication resistance which could be the primary reason for switching [52].

The odds of antipsychotic medication switch in patients who had a history of hospital admission in the past 1 year were 3 times higher than those patients without a history of a past admission. This finding is consistent with another study done in Ethiopia [21], and two studies done in the USA [53, 54]. Generally, hospital admissions in patients with schizophrenia may be indicative of treatment failure [55], the presence of side effects [38], or noncompliance [56] with the preceding antipsychotic regimen, which necessitate medication changes (switch) [17, 49].

The odds of an antipsychotic medication switch were 3.34 times higher in patients who were on typical antipsychotics than those patients on atypical antipsychotics. This finding is in line with the finding in the Netherlands [28]. Newer-generation antipsychotics have the potential to reduce relapse rates and treatment failure [57], which makes switching less common in atypical antipsychotics compared to typical antipsychotics. Furthermore, patients on conventional antipsychotics had persistent symptoms or unpleasant side effects which lead to the APs switch.

In the present study around 99.7% of switching was done by abrupt discontinuation of the first antipsychotics and starting the other antipsychotics. This finding is higher than a study done in Canada 33% [58], Netherlands 61% [59]. The reason why high abrupt switching in our setting might be due to a lack of expertise in choosing the appropriate antipsychotics based on patient-specific factors and pharmacokinetics and dynamics of antipsychotic drugs.

The rationality of the switching pattern and the switching techniques utilized in this study were assessed using the Australian antipsychotic switching tool. In the present study, the majority of patients were on chlorpromazine and they were switched to Respiridone. Sixty-four patients (32.7%) had switched from Chlorpromazine to Respiridone, 23 (11.2%) of the patients switched from Chlorpromazine to Haloperidol, and 8 (3.9%) of the patients were switched from Chlorpromazine to Olanzapine. All patients on the three patterns of switching abrupt switching technique were used. However, the abrupt switching technique is not the recommended technique due to the differences in adverse-effect profiles of the two drugs in the aforementioned three switching patterns. Switching is ideally done gradually rather than abruptly, to avoid symptom exacerbation and other rebound phenomena [60]. Cholinergic rebound, effects on blood pressure, and behavioral effects related to loss of sedation are expected when chlorpromazine has discontinued abruptly. In this regard, cross-titration is the recommended approach when the switching involves the discontinuation of chlorpromazine.

This study had several limitations, among them we included charts with complete information on the outcome and predictor variables and avoid cases with incomplete data on pertinent variables and the tool we used (Australian antipsychotic switching tool) is only used for switching from oral-oral/Depot-Depot formulations, and it cannot assess oral to depot or depot to oral formulations switching's, additionally, the tool contains a limited number of typical antipsychotics in the list.

## Conclusions and recommendations

In the present study, there is a high burden of antipsychotic medicine switch in schizophrenic patients in the follow-up clinics of five comprehensive specialized hospitals. Usually abrupt switching of antipsychotic medicine was practiced. Unavailability of the antipsychotic medicines was the most common reason to switch APs. Prescribers need to be careful about antipsychotic dosing and selecting the appropriate medication and switching strategies may help clinicians reduce discontinuation and unnecessary switch rate and thus achieve optimal clinical management.

## Data Availability

All data were included in the manuscript.

## Abbreviations

APs: Antipsychotics
AS: Antipsychotic switching
MARS: Medication Adherence Rating Scale
OSSS: Oslo Social Support Scale
CPZeq: Chlorpromazine Dose Equivalencies
LMICs: Low- and Middle-Income Countries
WHOADS: World Health Organization Disability Assessment Scale
